# Hydrazine Intercalation into 2D MoTe_2_ Field Effect Transistor as Charge Trapping Sites for Nonvolatile Memory Applications

**DOI:** 10.3390/nano15221721

**Published:** 2025-11-14

**Authors:** Li Yuan, Yongyu Wu, Haohui Ou, Di Wu, Yuhan Ji, Dianyu Qi, Wenjing Zhang

**Affiliations:** 1 ZJU-Hangzhou Global Scientific and Technological Innovation Center, Zhejiang University, Hangzhou 311200, China; yuanli2022@email.szu.edu.cn (L.Y.); yongyu.wu@zju.edu.cn (Y.W.); 2 International Collaborative Laboratory of 2D Materials for Optoelectronics Science and Technology, Institute of Microscale Optoelectronics, Shenzhen University, Shenzhen 518060, China; ouhaohui2016@email.szu.edu.cn (H.O.);; 3 College of Electronic Science and Technology, Xiamen University, Xiamen 361102, China; 4 Zhejiang Technology Innovation Center of CMOS IC Manufacturing Process and Design, Hangzhou 311200, China; 5 College of Electronic Engineering, Huainan Normal University, Huainan 232038, China

**Keywords:** hydrazine, charge trapping, interlayer doping, memory device

## Abstract

Driven by the demands of artificial intelligence, big data and the Internet of Things, non-volatile memory has become the cornerstone of modern computing. However, at present, most of the preparation processes are quite complex and have high requirements for the materials. Here, we discovered that hydrazine (N_2_H_4_) molecules can be efficiently intercalated into the MoTe_2_, acting as stable charge-trapping centers. This intercalation not only induces a controllable reversible polar conversion but also causes a huge hysteretic window (>60 V) lasting over one hour in air. Leveraging this giant hysteresis, we fabricated a simplified memory device. The device demonstrates a large erase/program current ratio of ~10^4^ and excellent retention characteristics. Our work pioneers the use of interlayer molecular intercalation for electronic modulation in 2D semiconductors, offering a new paradigm for developing memory devices with fabrication processes.

## 1. Introduction

To overcome the bottleneck of high energy consumption caused by the “memory wall” in conventional von Neumann architectures, neuromorphic computing has emerged as a promising pathway toward low-power artificial intelligence hardware by emulating the brain’s integration of computation and memory [1]. A central challenge lies in building non-volatile memory devices that mimic biological synapses, which must exhibit high energy efficiency, fast switching speeds, and long-term retention [2].

Among various emerging memory technologies, charge-storage devices based on two-dimensional (2D) materials—such as floating-gate and charge-trap memories—have shown considerable promise [3,4]. Their atomically smooth surfaces free of dangling bonds, combined with exceptional electrostatic control, offer an ideal platform for high-performance operation. For instance, MoS_2_-based floating-gate memory has demonstrated nanosecond-scale switching and data retention exceeding ten years [5], while the charge trap memory using graphdiyne achieved an ultra-low operating voltage of 30 mV, opening the way for extreme energy efficiency [6]. Devices using MoS_2_ as the channel can operate in extremely high-temperature environments [7]. However, most nonvolatile memory devices have a significant drawback—their manufacturing process is very complex. The floating gate requires multiple processing steps, while the ferroelectric structure has certain requirements for material properties.

2D semiconductors have shown great potentials in next-generation electronics for high-performance and short-channel field-effect transistors (FETs) due to their atomic thickness and high mobility [8,9,10,11,12]. For the application in complementary electronics, it is essential to effectively and precisely tune the electronic properties in a nondestructive manner [13]. In the past decade, various doping strategies for 2D semiconductors were developed, including surface charge transfer doping (SCTD), chemical modification and gas adsorption. For example, metal oxides’ deposition of Al_2_O_3_ and MgO induces n-type doping, and metal oxides’ deposition of MoO_3_ induces p-type doping effect on transition metal dichalcogenides (TMDs) transistors and situ oxygen passivation of sulfur vacancies achieved by performing chemical vapor deposition in atmospheric pressure conditions [14,15,16,17]. Chemical doping with a reducing reagent such as hydrazine hydrateand butyl lithium induces n-type doping, and doping with oxidizing reagent such as ozone, NO and NO_2_ induces p-type doping effect on TMDs [18,19,20,21,22]. Our group also reported several precise doping strategies such as in situ aluminum evaporation, laser irradiation, heating oxidation and van der Waals contact assembling and realized high-performance MoTe_2_ complementary inverters [23,24,25,26]. However, previous reported methods have their disadvantages. SCTD strategies require a capping dopant layer with several nanometers thickness on the surface of 2D semiconductors, seriously increasing the thickness of devices. Gas adsorption is based on the physical adsorption between gas molecules and 2D materials, which is too weak to maintain stable doping effects.

Given the abundance of active sites within the interlayers of 2D materials, and motivated by the success of ion-intercalated architectures in energy storage/conversion, we propose that interlayer modification surpasses surface modification in potential [27,28,29,30]. Nevertheless, the sub-1 nm confinement of these interlayer spaces poses significant challenges for molecular intercalation in 2D semiconductors, and interlayer molecular modification for modulating charge carrier of 2D FETs has yet to be reported.

In this work, we demonstrate that hydrazine molecules can intercalate and reside stably within the interlayers of MoTe_2_ flakes, serving as interlayer charge-trapping centers that effectively modulate charge transport. Compared with previously reported SCTD strategies, this hydrazine-induced interlayer modification not only enables efficient electrical doping but also significantly enlarges the electrical hysteresis of MoTe_2_. Importantly, the method is straightforward, minimally invasive and preserves the intrinsic properties of MoTe_2_. After just ten minutes of hydrazine treatment, a hysteresis window of up to 60 V is achieved, and the hysteresis behavior persists for over two hours without encapsulation. Leveraging this intercalation-induced hysteresis, we constructed a hydrazine-based charge-trapping MoTe_2_ non-volatile memory device, which exhibits an erase/program current ratio of ~10^3^ and excellent retention characteristics. Unlike conventional flash memory, this device operates without a floating gate or a dedicated charge-trapping layer, thereby circumventing complex microfabrication steps and avoiding additional device thickness. We believe this work opens new avenues for the application of 2D materials in memory technologies.

## 2. Materials and Methods

### 2.1. Materials

MoTe_2_ single crystals were purchased from HQ-graphene company. Hydrazine hydrate (10217-52-4, reagent grade, N_2_H_4_ 50–60%) was purchased from Sigma-Aldrich company (Burlington, MA, USA). Metals (Au, Pd, 99.99%) were purchased from ZhongNuo Advanced Material (Beijing) Technology Company (Beijing, China). Silicon wafer substrate (p-type doped, with 300 nm-thick SiO_2_) were purchased from Suzhou Crystal Silicon Electronic & Technology Company (Suzhou, China).

### 2.2. Methods

*Mechanical exfoliation of MoTe_2_ flakes*: Few-layer MoTe_2_ flakes were mechanically exfoliated by using Scotch tape and subsequently transferred onto precleaned silicon wafer substrate with 300 nm oxides. MoTe_2_ flakes with proper thickness were selected according to the optical contrast under optical microscopy.

*Fabrication of MoTe_2_ device*: Few-layer MoTe_2_ FETs were prepared by patterning electrodes via electron beam lithography (EBL, Raith, Dortmund, Germany, PIONEER Two) and evaporating Pd/Au (30/30 nm) as electrodes, which is the same as our previous work [23].

*Hydrazine vapor modification:* MoTe_2_ FET device was fixed on a sample holder and suspended in a 250 mL customized quartz chamber, and the chamber was sealed and heated to 75 °C, and then 0.5 mL hydrazine hydrate was added to the bottom of the chamber, and it immediately vaporized and filled the chamber. After the prepared MoTe_2_ FET was placed in the hydrazine atmosphere for 0.5 min for modification, the electrical performance was immediately tested. Then, the sample was further modified for 1.5 min (cumulatively 2 min), and the measurement was conducted again. Subsequently, the modification was continued until a total of 5 min had been reached, and the electrical performance was immediately tested at the end. Finally, when the total modification time reached 10 min, the electrical performance was measured for the final time. After ten minutes of modification and measurement of its electrical performance, the sample was kept in place and was not exposed to any external influences, as much as possible. The electrical performance of the device was measured at 5 min, 10 min, 140 min and 72 h.

*DFT Calculations*: All calculations were based on DFT, using the Vienna ab initio Simulation Package (VASP) code. The electron ion interaction was described with the projector-augmented wave method. The electron exchange and correlation energy were treated within the generalized gradient approximation in the Perdew–Burke–Enserch of formalism. The valence electrons were expanded in a plane-wave basis set with an energy cutoff of 460 eV. The hydrazine/MoTe_2_ was modeled by slab model with a vacuum thickness of 16 Å. The MoTe_2_ (001) surface was modeled by (2 × 2) supercell and the hydrazine was modeled by (1 × 1) supercell. The lattice mismatches of all heterojunctions were <5%. For the sampling of Brillouin-zone integrals, a Gamma-centered k-points grid of 5 × 5 × 1 was used. The convergence criterions of force and energy were set as 0.01 eV Å^−1^ and 10^−4^ eV, respectively.

### 2.3. Characterizations

Atomic force microscope was used to characterize the thickness of samples (Bruker Dimension ICON, Billerica, MA, USA). Raman spectra were performed with a 532 nm laser under ambient conditions (WITEC-α-300R, Ulm, BW, Germany). Electrical properties of devices were measured in a probe station (Lakeshore, TTPX, Westerville, OH, USA) with an equipped Keithley 4200 source measurement unit. X-ray photoelectron spectra were carried out on an ESCALab250Xi (Thermo Fisher Scientific Inc, Waltham, MA, USA) X-ray photoelectron spectroscope. *XAFS*: XAFS measurements at the Te *K*-edge were performed in fluorescence mode at the BL14W1 beamline of the Shanghai Synchrotron Radiation Facility (SSRF), Shanghai, China. The storage ring of SSRF was operated at 3.5 GeV with a maximum current of 210 mA.

*Cross-section Characterizations by FIB and STEM*: MoTe_2_ thin flakes were mechanically exfoliated from the bulk crystals and transferred onto the cleaned substrate. Then the chip is treated by hydrazine vapor for 5 min. To maintain the effect of hydrazine treatment, the hydrazine-modified sample is encapsulated by graphene and SiO_2_ for two steps. A thin graphene flake was first mechanically exfoliated and transferred onto the treated MoTe_2_ sample, and then a 15 nm-thick SiO_2_ was sputtered for further encapsulation. A cross-section of the hydrazine-modified MoTe_2_ sample was prepared by focused ion beam (FEI, Scios, Hillsboro, OR, USA). To protect the surface, a layer of 10 nm Pt and another layer of 1 µm Pt were successively deposited on the sample before FIB cutting. STEM was performed on Titan Cubed Themis G2 300 (Hillsboro, OR, USA).

## 3. Results and Discussion

**Figure 1:**
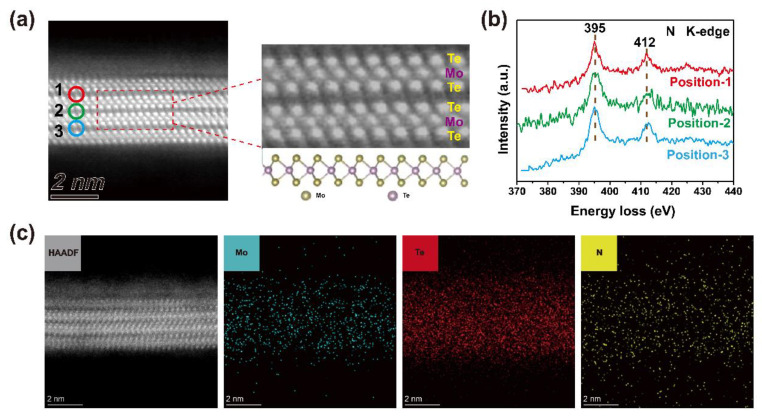
Structure and element composition characterization of MoTe_2_ after hydrazine treatment. (**a**) Cross-sectional STEM image of hydrazine-treated MoTe_2_. (**b**) N K-edge EELS spectra obtained at positions 1–3 in (**a**). (**c**) Cross-sectional STEM image and EDS elemental maps of Mo, Te, N of hydrazine-treated MoTe_2_.

Figure 1a presents the high-angle annular dark-field scanning transmission electron microscope (HAADF-STEM) image of MoTe_2_ after hydrazine treatment, showing the layered structure of MoTe_2_. Regular atomic arrangement of MoTe_2_ is consistent with typical 2H-MoTe_2_ without any lattice damage, indicating that hydrazine treatment is non-destructive and mild [31]. In order to study the distribution of hydrazine in the MoTe_2_ lattice, electron energy loss spectroscopy (EELS) is carried out using the energy loss of the K-shell edges of N element. Figure 1b shows the energy loss spectra of N K-edges at three positions in Figure 1a. Two peaks can be observed at 395 eV and 412 eV, corresponding to 1s-π* antibonding orbit bond and 1s-σ* antibonding orbit, respectively [32,33,34,35]. The π* and σ* features of N K-edge confirm that N elements are sp^2^ hybridized, which is consistent with the hybrid form of the nitrogen atom in the hydrazine molecule, showing that hydrazine exists in the interlayer of MoTe_2_ [34,36]. Figure 1c displays the EDS elemental maps of Mo, Te and N elements, also showing that nitrogen element distributes in the interlayer of MoTe_2_, demonstrating the intercalation of hydrazine into the interlayer of MoTe_2_. The result suggests that hydrazine treatment is an interlayer modification strategy, which is different from previous studies based on surface functionalization or deposition on the surface of 2D layered materials [37,38].

**Figure 2:**
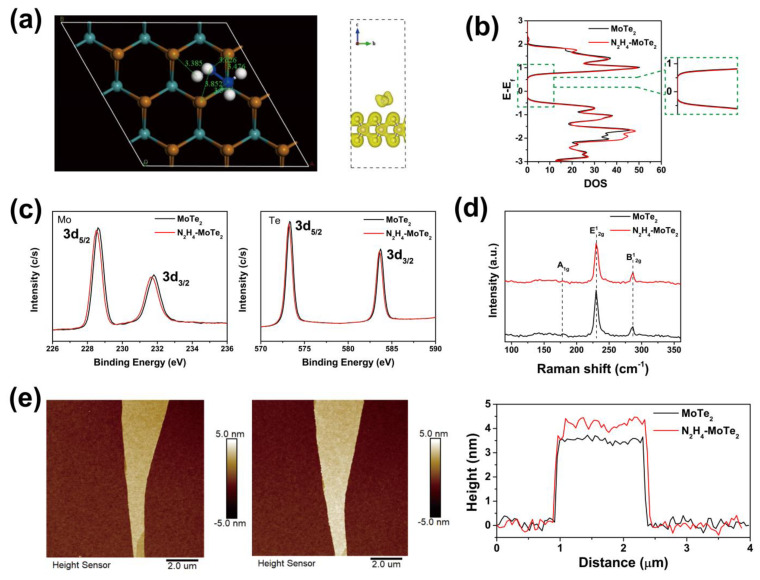
DFT calculation of hydrazine-treated MoTe_2_ and structural characterization from the pristine MoTe_2_ and hydrazine-treated MoTe_2_. (**a**) The state of individual hydrazine molecules adsorbed on the surface of MoTe_2_, inset picture illustrates the charge distribution of MoTe_2_ adsorbed by the hydrazine. (**b**) Comparison of DOS of pure MoTe_2_ and hydrazine-treated MoTe_2_. (**c**) XPS spectra of pristine MoTe_2_ and hydrazine-treated MoTe_2_ for Mo, Te. (**d**) Raman spectra of pristine MoTe_2_ and hydrazine-treated MoTe_2_. (**e**) Thickness images and surface topography of pristine MoTe_2_ and hydrazine-treated MoTe_2_.

In order to investigate the interaction between hydrazine molecules and MoTe_2_, we simulate the adsorption model between the hydrazine molecule and MoTe_2_ through density functional theory (DFT) and obtain the stable model with the lowest energy, as shown in Figure 2a. The nearest interatomic distances are over 3 Å, which is larger than the length of chemical bond. The right panel in Figure 2a shows the charge distribution of MoTe_2_ adsorbed by the hydrazine molecule, also indicating no chemical bond between the hydrazine molecule and MoTe_2_. Based on the above theoretic prediction, the adsorption between the hydrazine molecule and MoTe_2_ should be a van der Waals adsorption [39]. Figure 2b presents the energy levels of MoTe_2_ before and after adsorption by the hydrazine molecule; the bandgap of MoTe_2_ hardly changes, and no additional energy state is observed after adsorbing hydrazine molecule. The local density of states (DOS) of MoTe_2_ increases and the Fermi levels slightly move upward; this should be attributed to the electron transfer from the hydrazine molecule to MoTe_2_ [40].

To verify the interaction between hydrazine and MoTe_2_, we performed a series of experimental characterizations. Figure 2c displays XPS spectra of pristine MoTe_2_ and hydrazine-treated MoTe_2_. Two peaks of pristine MoTe_2_ at 228.6 eV and 231.8 eV can be observed, corresponding to Mo 3d_5/2_ and Mo 3d_3/2_, respectively [41,42]. For hydrazine-treated MoTe_2_, the peaks display a negative shift by 0.1 eV and 0.2 eV, respectively. Meanwhile, the same phenomenon can be observed in the XPS spectra of Te 3d signals. For the pristine MoTe_2_, two peaks of Te 3d_5/2_ and Te 3d_3/2_ locate at 573.3 eV and 583.7 eV and shift negatively by 0.1 eV and 0.1 eV, respectively, after treating by hydrazine [42]. The negative shift is attributed to the electron doping effect from hydrazine molecule. We believe this is due to the charge transfer that occurs between the hydrazine molecule and MoTe_2_, and a similar phenomenon was observed in our previous experimental study on Al-doped MoTe_2_ [26]. In addition, no new peak is observed in the spectrum, which proves that there is a kind of physical adsorption between MoTe_2_ and hydrazine, similar to van der Waals forces, and no new substances have been produced, as predicted by theoretical calculations [43].

Figure 2d presents the Raman spectra of pristine MoTe_2_ and hydrazine-treated MoTe_2_, showing three characteristic peaks at 171.2 cm^−1^ (A_1g_), 233.9 cm^−1^ (E^1^_2g_) and 289.6 cm^−1^ (B^1^_2g_), corresponding to 2H phase MoTe_2_ [44]. The spectrum of the hydrazine-treated sample is the same as that of the pristine MoTe_2_ without new peak or shift, indicating that hydrazine treatment contains the initial and intact lattice structure of pristine MoTe_2_, which is consistent with the analysis of EELS and XPS. Atomic force microscope (AFM) images (Figure 2e) show the surface of MoTe_2_ remains smooth and flat without any defects and cracks after hydrazine treatment. The thickness of MoTe_2_ flakes increases from 3.5 nm to 4.1 nm after hydrazine treatment. The thickness expansion may be caused by the intercalation of hydrazine to MoTe_2_.

**Figure 3:**
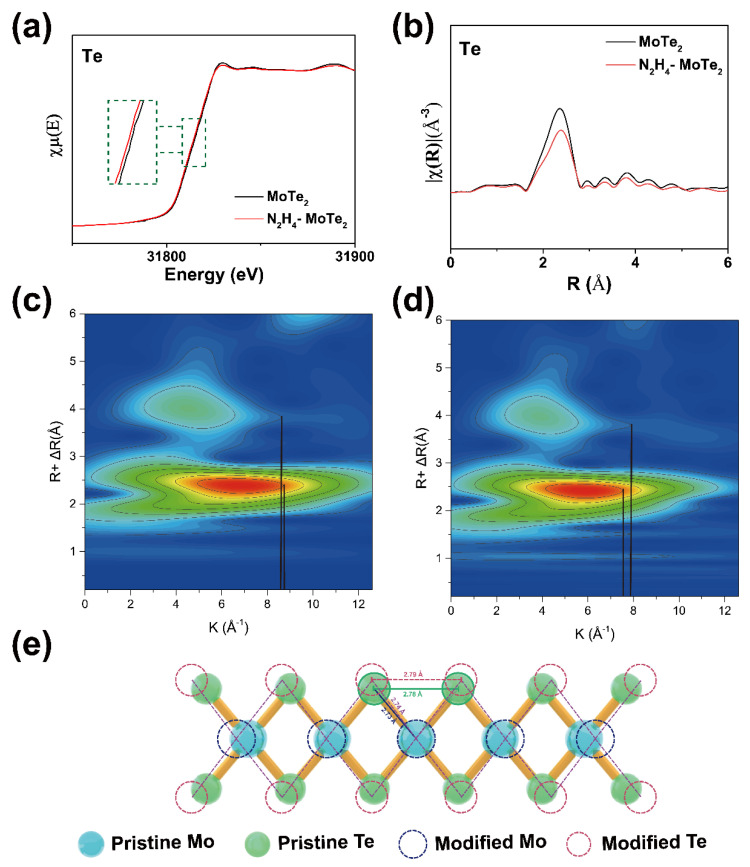
Surface-structural distortion induced by hydrazine molecular adsorption. (**a**) Te K-edge X-ray absorption near-edge structure spectra for MoTe_2_ and hydrazine-treated MoTe_2_. (**b**) The k^2^-weighted Fourier transform extended X-ray absorption fine structure spectra in R-space. (**c**,**d**) Corresponds to the wavelet transform of XANES (WT-EXAFS). (**e**) Surface structural distortion shown from a two-dimensional level.

X-ray absorption fine structure (XAFS) spectroscopy including X-ray absorption near edge structure (XANES) and extended X-ray absorption fine structure (EXAFS) is a powerful characterization for detecting the local atoms and electric structure around the selected absorbing atoms [45]. Figure 3a shows Te K-edge XANES spectra for MoTe_2_ and hydrazine-treated MoTe_2_. The absorption edge of hydrazine-treated MoTe_2_ almost overlaps with pristine MoTe_2_ with a lower energy than the pristine MoTe_2_, indicating the weaker bonding energy of Te elements of hydrazine-treated MoTe_2_ [46]. The similar shape of absorption edge further suggests that the same valence state of Te elements is present in pristine MoTe_2_ and hydrazine-treated MoTe_2_, proving that there is no chemical bond between hydrazine and MoTe_2_ [47]. The result is consistent with the analyses of DFT and XPS. Figure 3b displays corresponding Fourier transform (FT) of the corresponding k^2^-weighted Te edge EXAFS spectra as a function of the non-phase-corrected radial distance R [45]. The Mo-Te and Te-Te peak of hydrazine-treated MoTe_2_ is significantly decreased and shifted to high R. For the pristine MoTe_2_ nanosheet, the distance of Mo-Te and Te-Te are 2.73 Å and 2.78 Å, respectively. In comparison with pristine MoTe_2_, the hydrazine-treated MoTe_2_ has a longer bond length by 0.01 Å and a lower coordination number. An analysis of the local structure of MoTe_2_ and treated MoTe_2_ is performed with the fitted structural parameters summarized in Table 1.

The crystal structure of MoTe_2_ exhibits slight distortion after treatment with hydrazine. Fourier transform (FT) image reveals that hydrazine-treated MoTe_2_ has a longer radial distance (R) and lower coordination number (or structure disorder degree) with decreased peak area [47]. The special structure of the hydrazine can explain the local structural modulation after treatment. The electronegativity of nitrogen atom is stronger than that of hydrogen atom, which gives the N-H bond polarity and creates a non-uniform distribution of electron density around the N-N bond. The hydrazine molecule has an extremely strong polarity of 1.83 Debye due to the structural asymmetry [48,49]. In addition, the overall integrity of the crystal structure remained intact, consistent with previous EELS analyses. The strong reducibility of the hydrazine molecule guarantees the integrity of the crystal structure of MoTe_2_ [48], and the decreasing of coordination number of hydrazine-treated MoTe_2_ is due to the strong polarity, too. The distortion caused by hydrazine results in the increase in the structure disorder degree, which makes the coordination number decrease [47,48].

Additionally, wavelet transformation (WT) images can effectively illustrate this phenomenon. Unlike the two-dimensional information obtained from the R space through Fourier transform, WT analysis can combine the R space and k space to obtain three-dimensional information [50]. The horizontal axis of the wavelet transformation represents the wave vector, which plays a crucial role in distinguishing different types of coordinating atoms [50,51]. With the smaller atomic number, the corresponding x-coordinate (k) shifts to the lower area [52]. As observed in Figure 3c,d, the contour plot has shifted leftward, indicating the change in the coordination atom. We infer that the phenomenon can be attributed to the change in the coordination environment of Te atoms resulting from the charge transfer from hydrazine molecules to MoTe_2_. The fitting results of two samples is drawn in Figure 3e.

**Figure 4:**
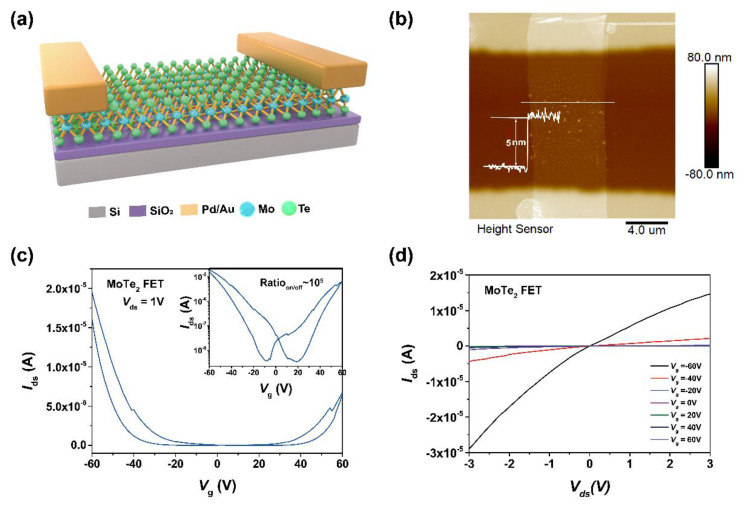
Basic characterization of MoTe_2_ FET devices. (**a**) Illustration the structure of the MoTe_2_ FET. (**b**) AFM image and thickness profile of the channel in the MoTe_2_ device. (**c**) Transfer cure (*I*_ds_ − *V*_g_) of MoTe_2_ FET at *V*_ds_ = 1 V on liner scale. Inset: Transfer curve of MoTe_2_ FET at *V*_ds_ = 1 V on logarithmic scale. (**d**) Output curve (*I*_ds_ − *V*_ds_) of the same device with *V*_g_ ranging from −60 V to 60 V.

To investigate the effect of hydrazine treatment on the electrical property of MoTe_2_ nanosheet, a field effect transistor (FET) based on MoTe_2_ was fabricated and then treated by hydrazine vapor. Figure 4a presents a three-dimensional perspective of the FET, in which MoTe_2_ serves as the channel, palladium/gold (Pd/Au) as source and drain, and SiO_2_/P^+^-doped Si as back gate. First, mechanical-exfoliated MoTe_2_ is selected on PDMS under the optical microscopy and transferred onto cleaned Si substrates with 300 nm SiO_2_. The Pd/Au electrodes as metal contacts are made onto the MoTe_2_ nanosheet by metal lift-off process. Figure 4b presents the AFM image of the as-presented FET. The line profile along the white solid line in Figure 4b shows that the thickness of MoTe_2_ nanosheet is 5 nm, corresponding to 7 layers [53]. The device presents a typical ambipolar transfer characteristic (*I*_ds_ − *V*_g_) as illustrated in Figure 4c. Applying a gate voltage (*V*_g_) from −60 V to 60 V, the source–drain current (*I*_ds_) increases from OFF to ON state along with negative and positive sweeping of *V*_g_, corresponding to the electron and hole transport, respectively. The current at *V*_g_ = −60 V is higher than that at *V*_g_ = 60 V, indicating it is a p-type dominated ambipolar FET. The transfer curve shows an on/off ratio is ~5.6 × 10^3^ and small electrical hysteresis. Figure 4d shows the linear output curve of the device, revealing the good contact between Pd-Au metal electrodes and MoTe_2_.

In order to characterize the influence of hydrazine vapor on the electrical performance of MoTe_2_ FET, we first characterized the electrical properties of the device at its initial state. Then, we exposed the device to hydrazine vapor for 30 s and immediately measured its electrical properties afterwards. Then, the duration for additional modification was extended to two minutes. The electrical properties were measured again, and this process was repeated until the total modification time reached ten minutes. Subsequently, in situ desorption tests were conducted on the device.

**Figure 5:**
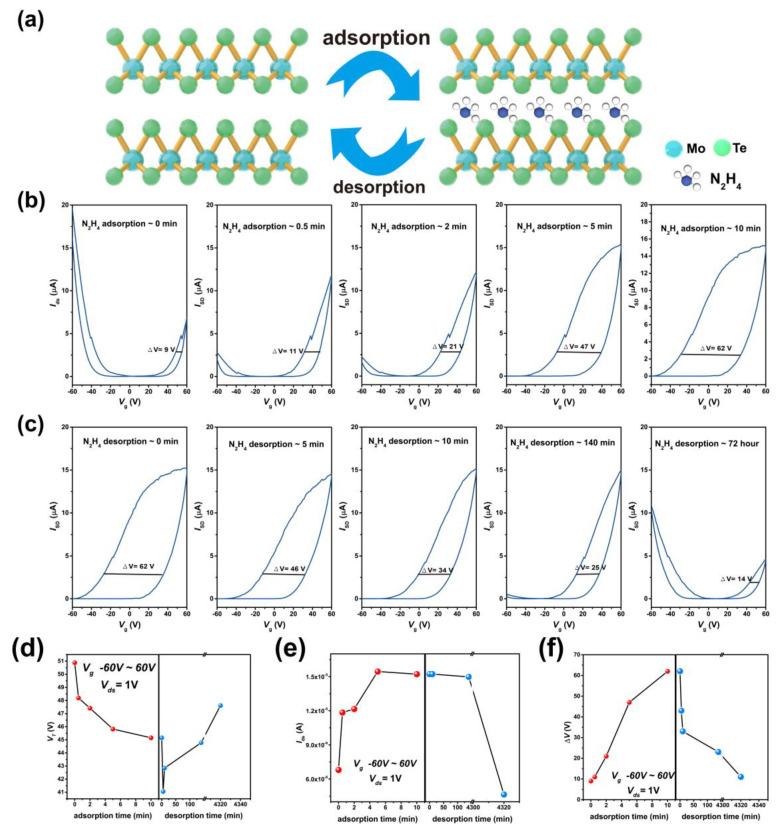
Electrical characterization of the intercalation and release process of hydrazine treated. (**a**) The process of intercalation and release. (**b**) Transfer curves of hydrazine-treated device as adsorption time of hydrazine vapor molecules increases. (**c**) Transfer curves of hydrazine-releasing device as desorption of hydrazine vapor molecules time increases. (**d**) Changes in the threshold voltage with the adsorption and desorption of hydrazine vapor molecules. (**e**) Changes in current with the adsorption and desorption of hydrazine vapor molecules. (**f**) Changes in hysteresis window with the adsorption and desorption of hydrazine vapor molecules.

Then, we systematically investigated the influence of hydrazine adsorption and desorption on the electrical properties of MoTe_2_ FETs. Figure 5a–c schematically illustrates the experimental process and the corresponding evolution of the transfer characteristics. The pristine device exhibits a hole-dominated ambipolar transport behavior. After 30 s of hydrazine treatment, the transfer curve evolves into an n-type dominated ambipolar characteristic. As the treatment time increases, the electron branch drain current rises while the hole branch current declines, accompanied by a clear negative shift in the threshold voltage of the electron branch. This polarity reversal can be ascribed to the enhanced electron concentration in MoTe_2_ resulting from the strong electron-doping effect of hydrazine, ref. [54] in agreement with our DFT calculations. The drain current saturates at approximately 15 μA after 5–10 min of hydrazine exposure.

In addition to the transition from p to n-type dominance, a pronounced electrical hysteresis emerges after hydrazine treatment. As depicted in Figure 5b, the hysteresis window of the pristine device is 9 V. It increases slightly to 11 V after 30 s of treatment and expands significantly with prolonged exposure, reaching 62 V after ten minutes. This widening hysteresis is attributed to the charge-trapping effect of hydrazine molecules, a phenomenon previously observed in gas-adsorbed 2D channels [55]. The hysteresis can be explained by an electron trapping/detrapping model [56]. When the gate voltage sweeps from −60 V to 60 V, the MoTe_2_ channel is initially electron-depleted; electrons transferred from adsorbed hydrazine molecules fill trap states, reducing carrier depletion and shifting the threshold voltage negatively. Conversely, during the reverse sweep from 60 V to −60 V, electrons are initially abundant in the channel; some are transferred to hydrazine molecules and trapped, enhancing carrier depletion and causing a positive threshold voltage shift.

Notably, the hysteresis is reversible and diminishes with hydrazine desorption. Figure 5c presents the transfer characteristics measured at different desorption times. Initially, the device shows n-type behavior with a hysteresis window of ~62 V. Over 140 min, the window gradually narrows to 25 V, while the drain current remains stable around 15 μA. After 72 h of desorption, the hysteresis further reduces to 14 V, and the transfer characteristic reverts to a p-type dominated ambipolar shape, with hole and electron branch currents of 10 μA and 4 μA, respectively—closely resembling the pristine state.

To quantitatively track the electrical evolution during adsorption and desorption, key parameters including threshold voltage (*V*th), electron branch current (*I*_n_), and hysteresis window (ΔV) were extracted from the transfer curves, as summarized in Figure 5d–f. After hydrazine adsorption, *V*th shifts from 51 V to 44 V and recovers to 48 V after desorption; *I*_n_ increases from ~6.8 μA to 15.2 μA and returns to 4.6 μA; ΔV expands from 9 V to 62 V and eventually recovers to 14 V. We calculated some parameters based on the obtained curve. Based on the thickness and width of the material, we calculated that the electron current densities of pristine MoTe_2_ and treated MoTe_2_ were 0.086 mA/µm^2^ and 0.2 mA/µm^2^, respectively. We separately calculated the carrier mobility of MoTe_2_ before and after doping. The mobilities of electrons and holes before modification were 2.33 cm^2^ V^−1^ s^−1^ and 2.95 cm^2^ V^−1^ s^−1^. After doping, the mobilities of electrons and holes before modification were 2.47 cm^2^ V^−1^ s^−1^ and 0.02 cm^2^ V^−1^ s^−1^. The mobility of the treated holes is significantly reduced, while the mobility of electrons increases. In addition, we also calculated the charge current density of the device. The charge current density before modification is 1.45 × 10^11^ cm^−2^ and the charge current density after modification is 9.34 × 10^11^ cm^−2^. Clearly, the electron current density of MoTe_2_ increased after doping. This is because there is a charge transfer between the hydrazine and MoTe_2_, increasing the carrier density in the channel. The results indicate hydrazine treatment is a reversible strategy, which can induce n-type doping and a significant hysteresis effect on the MoTe_2_ FETs.

Bartolomeo’s group studied the hysteresis effect of oxygen, nitrogen, hydrogen, argon and methane gas adsorbed on back gated MoS_2_ FETs on 300 nm SiO_2_/Si substrate, in which the hysteresis window of methane adsorption is the largest (32 V), and that of argon adsorption is the smallest (5 V). They found the hysteresis effect shows strong correlation with the adsorption energy (*E*_ads_ = *E*_total_ − *E*_2D_ − *E*_gas_), and stronger gas adsorption can induce a larger hysteresis window on device performance. In this study, we observed that hydrazine adsorption can induce a much larger hysteresis window (62 V), suggesting the interaction of hydrazine with MoTe_2_ is extremely strong. This should be attributed to the intercalation of hydrazine into the interlayer of MoTe_2_, as the interlayer structure provides abundant adsorption sites and confines the adsorbed hydrazine molecules (supported by above EELS results), ensuring stable molecular adsorption of hydrazine.

**Figure 6:**
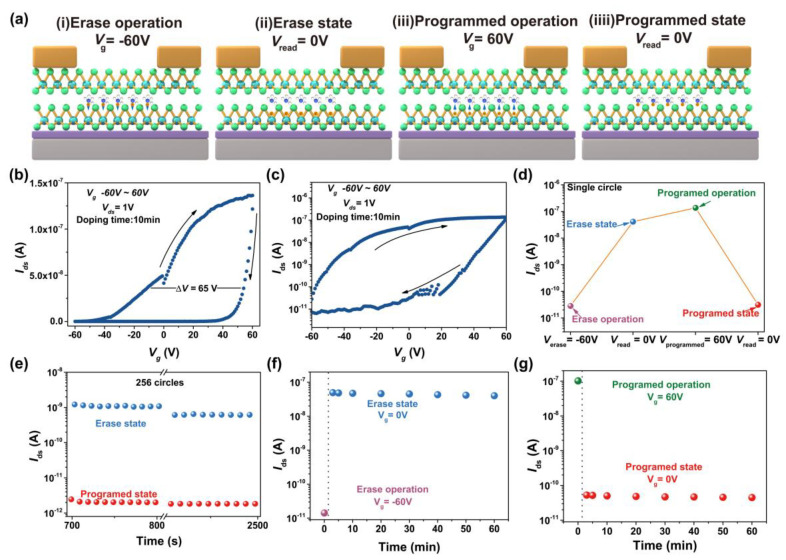
Memory principle and process of the hydrazine-treated MoTe_2_ FET. (**a**) Visualizing the programmed and erase principle. (**b**) Transfer curve of MoTe_2_ FET with treating by hydrazine vapor molecules for 10 min. (**c**) Logarithmic plot of the transfer curve (**b**). (**d**) Single circle of memory process. (**e**) 256 circles of memory process. (**f**) Data retention capability of erase operation within one hour. (**g**) Data retention capability of programmed operation within one hour.

Based on the above hysteresis characteristics, we further investigated the application of this modification method on MoTe_2_ FETs towards memory device. Figure 6a illustrates the memory mechanism of hydrazine-modified MoTe_2_ FETs. (i) Erase operation: A negative bias of −60 V is applied to the back gate electrode, a positive bias of 1 V is applied to drain electrode, and source electrode is grounded. (ii) Read after erase operation: A positive bias of 1 V is applied to drain electrode, and source electrode and back gate electrode are grounded, the device is in “ON” state. (iii) Program operation: a positive bias of 60 V is applied to the back gate electrode, a positive bias of 1 V is applied to drain electrode, and source electrode is grounded. (iv) Read after program operation: A positive bias of 1 V is applied to drain electrode, source electrode and back gate electrode are grounded, and the device is in “OFF” state. Figure 6b,c shows the transfer curve of MoTe_2_ FETs device after 10 min treated by hydrazine, which presents a hysteretic window of ~65 V. Figure 6d presents a process of the single erase/program circle. The read currents after erasing and program operation are ~5 × 10^−8^ A and ~2 × 10^−11^ A, respectively, giving a current ratio of 10^3^. Cycling test shows the device is stable after 256 erase/program cycles (Figure 6e), and retention test shows the device performance stably maintains for one hour (Figure 6f,g), showing the hydrazine vapor treatment is a robust strategy for realizing a hysteresis memory device based on MoTe_2_.

## 4. Conclusions

In summary, we studied hydrazine-modified 2D layered MoTe_2_ flakes and found that hydrazine molecules can enter and stably exist in the interlayer of 2D layered MoTe_2_ flakes. Raman spectra and AFM show that the modification is non-destructive to MoTe_2_ flakes, and DFT calculations and XPS results show there is no chemical bond between MoTe_2_ and hydrazine, indicating the adsorption mode is physical or van der Waals adsorption. XAFS shows the hydrazine modification induces slight structure distortion of MoTe_2_ lattice. In addition, we also studied the effect of hydrazine interlayer modification on few-layered MoTe_2_ FETs and found that hydrazine adsorption induces n-type doping and strong hysteresis for the transfer characteristics of the device. There is a charge transfer between the hydrazine vapor molecules and MoTe_2_, which leads to an increase in the current at the N branch. Based on the novel interlayer modification strategy, we developed a charge-trapping, molecular-based MoTe_2_ memory device, which shows an erase/program current ratio of ~10^3^ and good electronic retention ability. This research is expected to promote the development of 2D electronic devices in the memory field.

## Figures and Tables

**Table 1:** Mo-Te and Te-Te bond length information fitted according to XAFS.

Sample	Shell	CN	R (Å)	σ^2^ (10^−2^ Å^2^)	∆E_0_ (eV)	r-Factor (%)
MoTe_2_	Te-Mo	4.9	2.73	0.7	−18.2	1.0
	Te-Te	2.9	2.78	0.5	−18.2	
Treated-MoTe_2_	Te-Mo	3.9	2.74	0.7	−17.4	1.3
	Te-Te	2.2	2.79	0.5	−17.4	

## Data Availability

The data that support the findings of this study are available from the corresponding author upon reasonable request.
